# 

**Published:** 1887-01

**Authors:** 


### CONTENTS:

ARTICLE I—Our Duty to our Profession. By E. P. Beadles, D. D. S... 385
" II—Notes on the Decay of Human Teeth. By Prof. W. D. Miller 390
" III—The Period of Puberty and the Teeth. By Dr. D. T. Nelson. 394
" IV—A Criticism of the Herbst Method of Filling Teeth. By W. W. Allport, D. D. S .......... 399
" V—Drumine—The New Local Anæsthetic.......... 405
" VI—A Superior Wisdom Tooth Discharged from the Nasal Passages; with Remarks. By John S. Marshall, M. D........ 408
" VII—A Logical Inference. By Fred. A. Bellamy .......... 411
"VIII—Cancrum Oris.......... 414
" IX—The Odontological Society of Great Britain.......... 415
The Louisiana State Dental Association.......... 423

### EDITORIAL, ETC.

University of Maryland, Dental Department, Annual Commencement for 1887.......... 424
The Pasteur Methods .......... 424

### MONTHLY SUMMARY.

Fatal Result of Peroxide of Hydrogen Injections.......... 425
How to Become a Good Dentist.......... 426
Filling Proximal Cavities.......... 427
Personal Cleanliness and Unpleasant Odors, and Disease .......... 428
Physicians Extracting Teeth.......... 431
The Most Popular Medicines.......... 432
Dentists Damaged by the Earthquake at Charleston.......... 432

